# Search for and functional annotation of multi-domain PLA2 family proteins in flatworms

**DOI:** 10.18699/vjgb-24-93

**Published:** 2024-12

**Authors:** M.E. Bocharnikova, I.I. Turnaev, D.A. Afonnikov

**Affiliations:** Institute of Cytology and Genetics of the Siberian Branch of the Russian Academy of Sciences, Novosibirsk, Russia Novosibirsk State University, Novosibirsk, Russia Kurchatov Genomic Center of ICG SB RAS, Novosibirsk, Russia; Institute of Cytology and Genetics of the Siberian Branch of the Russian Academy of Sciences, Novosibirsk, Russia Kurchatov Genomic Center of ICG SB RAS, Novosibirsk, Russia; Institute of Cytology and Genetics of the Siberian Branch of the Russian Academy of Sciences, Novosibirsk, Russia Novosibirsk State University, Novosibirsk, Russia Kurchatov Genomic Center of ICG SB RAS, Novosibirsk, Russia

**Keywords:** phospholipase A2, flatworms, multi-domain proteins, parasitism, phylogeny, domain structure, фосфолипаза А2, плоские черви, многодоменные белки, паразитизм, филогения, структура доменов

## Abstract

The phospholipase A2 (PLA2) is a superfamily of hydrolases that catalyze the hydrolysis of phospholipids and play a key role in many molecular processes in the cells and the organism as a whole. This family consists of 16 groups divided into six main types. PLA2 were first isolated from venom toxins and porcine pancreatic juice. The study of these enzymes is currently of great interest, since it has been shown that a number of PLA2 are involved in the processes of carcinogenesis. PLA2 enzymes were characterized in detail in model organisms and humans. However, their presence and functional role in non-model organisms is poorly understood. Such poorly studied taxa include flatworms, a number of species of which are human parasites. Several PLA2 genes have previously been characterized in parasitic flatworms and their possible role in parasite-host interaction has been shown. However, no systematic identification of the PLA2 genes in this taxon has been carried out. The paper provides a search for and a comparative analysis of PLA2 sequences encoded in the genomes of flatworms. 44 species represented by two free-living and 42 parasitic organisms were studied. The analysis was based on identification of orthologous groups of protein-coding genes, taking into account the domain structure of proteins. In flatworms, 12 of the 13 known types of animal A2 phospholipases were found, represented by 11 orthologous groups. Some phospholipases of several types fell into one orthologous group, some types split into several orthogroups in accordance with their domain structure. It has been shown that phospholipases A2 of the calcium-independent type, platelet-activating phospholipases from group G8 and lysosomal phospholipases from group G15 are represented in all large taxa of flatworms and the vast majority of the species studied by us. In free-living flatworms PLA2 genes have multiple copies. In parasitic flatworms, on the contrary, loss of genes occur specifically in individual taxa specifically for groups or subfamilies of PLAs. An orthologous group of secreted phospholipases has been identified, which is represented only in Digenea and this family has undergone duplications in the genomes of opisthorchids. Interestingly, a number of experimental studies have previously shown the effect of Clonorchis sinensis proteins of this orthogroup on the cancer transformation of host cells. Our results made it possible for the first time to systematically identify PLA2 sequences in flatworms, and demonstrated that their evolution is subject to gene loss processes characteristic of parasite genomes in general. In addition, our analysis allowed us to identify taxon-specific processes of duplication and loss of PLA2 genes in parasitic organisms, which may be associated with the processes of their interaction with the host organism.

## Abstract (ru)

Фосфолипазы типа A2 (ФА2) – это семейство гидролаз, которые катализируют процесс гидролиза фосфолипидов, играя ключевую роль во многих молекулярных процессах при функционировании клеток и организма в целом. Данное семейство подразделяется на 16 групп, объединенных в шесть основных типов.Впервые ФА2 были выделены как цитотоксины яда у змей и ферменты панкреатического сока у свиней. Изучение этих ферментов в настоящее время вызывает большой интерес, поскольку было показано, что ряд ФА2 участвует в процессах канцерогенеза. Наиболее хорошо изучены ферменты ФА2 у модельных организмов и человека. Однако их наличие и функциональная роль у немодельных организмов изучены слабо. К таким малоизученным таксонам относятся плоские черви, ряд видов которых является паразитами человека. У паразитических плоских червей ранее было охарактеризовано несколько генов ФА2 и показана их возможная роль во взаимодействии «паразит–хозяин». Но систематической идентификации генов ФА2 у этого таксона не проведено. В работе осуществлены поиск и сравнительный анализ последовательностей ФА2, кодируемых в геномах плоских червей. Исследовано 44 вида, представленных 2 свободноживущими и 42 паразитическими организмами. Анализ выполнен на основе поиска ортологических групп белок-кодирующих генов с учетом доменной структуры белков. У плоских червей обнаружено 12 из 13 известных типов фосфолипаз А2, имеющихся в 11 ортологических группах. Часть фосфолипаз нескольких типов попала в одну ортологическую группу, часть типов распалась на несколько ортогрупп в соответствии с особенностями доменной структуры. Показано, что ФА2 кальций-независимого типа, ФА2 тромбоцитарно-активирующего типа групп G8 и лизосомальные ФА2 группы G15 представлены во всех крупных таксонах плоских червей и в большинстве изученных нами видов. Для генов, кодирующих ферменты у свободноживущих червей, наблюдается множественное число копий. У паразитических плоских червей, наоборот, происходит потеря основной части генов специфически по отношению как к отдельным таксонам, так и к отдельным группам/подсемействам ФА2. Обнаружена ортологическая группа секретируемых фосфолипаз, которая среди паразитов имеется только у дигенетических сосальщиков, при этом в геномах описторхид это семейство подверглось дупликациям. Интересно, что ранее в ряде экспериментальных работ показано влияние белков Clоnorchis sinensis этой ортогруппы на раковую трансформацию клеток организма-хозяина. Наши результаты дали возможность впервые систематически идентифицировать последовательности ФА2 у плоских червей и продемонстрировали, что их эволюция подвержена процессам потерь генов, характерных в целом для геномов паразитов. Кроме того, наш анализ позволил выявить таксон-специфические процессы дупликаций и потерь генов ФА2 у паразитических организмов, которые могут быть связаны с процессами их взаимодействия с организмом хозяина.

## Introduction

The protein family of phospholipases A2 (PLA2) is a group of hydrolases that catalyze the hydrolysis of phospholipids, playing a key role in the functioning of cells and the organism as a whole (Filkin et al., 2020; Murakami et al., 2020). Phospholipases A2 are known to be the main components of venom toxins in snakes (Bitar et al., 2021), insects (Bitar et al., 2021), predatory invertebrates, for example, arachnids (Salabi, Jafari, 2024) or mollusks (McIntosh et al., 1995). Phospholipases A2 from snake venom hydrolyze phospholipids of cell membranes, which leads to cell destruction, release of arachidonic acid and activation of inflammatory processes. Their effects can also lead to more serious pathogenic effects, including damage to the nervous system (Bitar et al., 2021), which demonstrates the multiplicity of their functions (Gutiérrez, Lomonte, 2013).

The PLA2 family is divided into 16 groups (Dennis et al., 2011), united into six main types: secreted, cytosolic, calcium-independent, platelet-activating factors, lysosomal and adipospecific (Murakami et al., 2020). The main molecular functions of PLA2 include lipid cleavage, fatty acid remodeling, and interaction with phospholipids of lysosomes and adipose tissue (Mouchlis, Dennis, 2022). In animals, these enzymes are involved in a large number of important processes related to antibacterial, antiviral, immune and antiinflammatory activities (Dennis et al., 2011).

The antiparasitic properties of phospholipases A2 are also known (Teixeira et al., 2022). Currently, these proteins are of great interest due to the fact that the impairment of lipid metabolism regulated by PLA2 often leads to various diseases, including carcinogenesis (Turnaev et al., 2022). Secreted PLA2 have increased expression in malignant tumors of organs such as the stomach (Scott et al., 2010), lungs (Park et al., 2012), intestines (Murase et al., 2017) and liver (Shang et al., 2017).

PLA2 are ancient genes and are found in all taxa of living organisms – bacteria, protists, archaea, animals, fungi and plants (Nevalainen et al., 2012). Their evolutionary analysis allows to consider in more detail the functional features of these proteins, to clarify their role in the most important biological processes (Murakami et al., 2020; Turnaev et al., 2022). PLA2 enzymes have been most well studied in model organisms and humans. However, their presence and functional role in non-model organisms have been poorly studied. Such poorly studied taxa include flatworms, a number of species of which are human parasites

Flatworms (Platyhelminthes) are one of the oldest groups of multicellular animals. Their origin goes back to the early stages of the evolution of multicellular organisms. Studies by B. Egger et al. (2015) show that flatworms appeared more than 500 million years ago, during the Cambrian period, making them one of the first animals with an organized tissue structure. Along with mollusks (Mollusca) and annelids, they belong to a broader group, Lophotrochozoa (Egger et al., 2015; Laumer et al., 2015). At the same time, flatworms are often considered as a sister group to mollusks (Laumer et al., 2015), which emphasizes their close evolutionary relationship. The importance of studying the biology of flatworms is due to the fact that most of their species are parasites – the main agents of helminthic diseases transmitted through infected fish, affecting a significant number of people.(On the state of sanitary and epidemiological welfare of the population in the Russian Federation in 2014: a state report. Moscow: Rospotrebnadzor, 2015, vol. 206) Numerous studies have shown that long-term infections such as opisthorchiasis, schistosomiasis and similar helminthiasis can lead to serious consequences for the health of the host organism (Carbonell et al., 2021; Ogorodova et al., 2015; Pakharukova et al., 2019a), including the development of cancer (Pakharukova et al., 2019b; Mordvinov et al., 2021).

In parasitic flatworms, phospholipase A2 is widely present in excretory secretory products (ESP), which are secreted to affect the host (Wang et al., 2014), indicating the potential pathogenic effects of these enzymes on the host body. For example, a number of studies have experimentally shown that phospholipases A, C, and D of the parasitic flatworm Clonorchis sinensis are associated with fibrosis in the host (Hu et al., 2009). It has also been shown that phospholipases A2 of group 3 of C. sinensis are involved in the processes of carcinogenesis in host cells (Shang et al., 2017). However, currently there is only scattered information about phospholipases A2 in flatworms and their representation in genomes. Their functions in parasites are poorly described. This highlights the need for a deeper analysis and annotation of the functions of phospholipases A2 in flatworms, including parasitic worms, in order to better understand their role in pathogenesis and develop effective methods to combat helminthic infections.

In this work the structure, functions and evolution of phospholipases A2 in flatworms were studied. Identification of the phospholipase A2 protein sequences in flatworms was performed, and they were divided into orthogroups. Phylogenetic analysis of sequences from the orthogroups was carried out. Domain strictures and putative functions of PLA2 enzymes were analyzed.

## Materials and methods

**Fig. 1.**
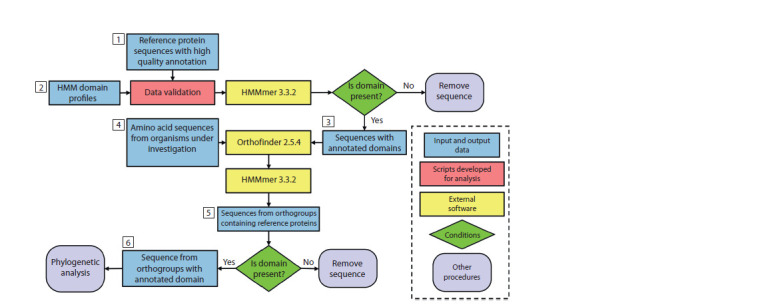
Block diagram of the OrthoDom computing pipeline. Block designations are shown in a dotted rectangle on the right.

**The OrthoDom computational pipeline for the identification of orthologous groups of proteins taking into account the domain structure. ** To identify PLA2 orthologous groups in flatworms, taking into account the domain structure, we used information on reference sequences of well-annotated PLA2 in model animals and the OrthoDom computational pipeline. The scheme is shown in Figure 1.

The OrthoDom pipeline allows to search for sequences of families of multidomain proteins in protein sequences encoded in the genomes of organisms under study based on orthology and domain analysis. As input data (marker 1 in Figure 1), sequences of the family of multidomain proteins with high-quality annotation (as a rule, identified and annotated in model organisms) are used (reference sequences). For reference sequences, lists of functional domains that they include are specified. For these domains, the corresponding HMM profiles (marker 2) are extracted from the Pfam 33.1 1 On the state of sanitary and epidemiological welfare of the population in the Russian Federation in 2014: a state report. Moscow: Rospotrebnadzor, 2015, vol. 206. database (Mistry et al., 2021). Further, using the hmmsearch program of the HMMer 3.3.2 package (Eddy, 2011), validation of reference proteins is carried out for the presence of these domains (marker 3), since for some of them domains may be fragmented or absent.

Another set of input data is the amino acid sequences (proteomes) of the studied organisms (usually non-model ones), in which it is required to determine the orthologs of the reference proteins (marker 4). Orthologous groups for amino acid sequences of reference proteins and proteins of the studied organisms were determined by the OrthoFinder v. 2.5.4 program (Emms, Kelly, 2019). The orthologous groups of interest are identified (marker 5) by the presence of reference sequences. Sequences were additionally checked for the presence of specified domains. The sequences of orthologs of reference proteins identified in this way (marker 6) were further processed for phylogeny reconstruction by the IQ-TREE program (Nguyen et al., 2015). Phylogenetic trees were visualized using the web version of the iTOL program (Letunic, Bork, 2024).

**Reference sequences of phospholipases A2 and their functional domains. **To identify phospholipases A2 in flatworms, we used well annotated sequences of vertebrate phospholipases classified by type in a number of previous works. These proteins were considered as reference and were used to determine the type of phospholipases in orthologous groups of flatworm proteins. The sample of reference proteins is based on the PLA2 sequences from the work (Huang et al., 2015) (9 types of phospholipases in humans and some vertebrates). These sequences were supplemented with sequences from the NCBI database identified on the basis of homology using BLASTP (Turnaev et al., 2022). According to the classification of phospholipases A2 proposed by M. Murakami et al. (2020), out of a total of 16 groups of phospholipases of living organisms, the reference sample included phospholipases of 13 groups, since groups of phospholipases A2 11, 13 and 14 are present only in plants (Murakami et al., 2020). As a result, the reference sample of phospholipases A2 included 13 groups of PLA2 from 15 vertebrate taxa. The list of reference sequences from the articles by I.I. Turnaev et al. (2022) and Q. Huang et al. (2015), the type of phospholipase, the species name of the organism, and the identifier used in this work are given in Supplementary Material 12. The list of key domains of these proteins and their HMM models is given in Supplementary Material 2.


Supplementary Materials are available in the online version of the paper: http://vavilov.elpub.ru/jour/manager/files/Suppl_Bocharnikova_Engl_28_8.pdf


Since phospholipases A2 contain not only PLA2 domains, but also other characteristic domains (Dennis et al., 2011), they were also identified after the identification of orthologs. The list of protein domains considered is provided in Supplementary Material 3.

**Sources of genomic data.** We studied the sequences of protein-coding genes from the genomes of flatworms of 44 species represented by two free-living and 42 parasitic organisms. The amino acid sequences encoded by mRNAs of the corresponding genes presented in the Wormbase Parasite 18.0 database (Howe et al., 2017) were analyzed. These species include the main taxa of flatworms: the class of digenetic flukes (Digenea), the class of tapeworms (Cestoda), the class of monogenetic flukes (Monogenea) and the class of ciliated worms (Turbellaria) (Brusa et al., 2020). Among the listed classes, the latter is a class of free-living worms, representatives of all other classes are obligate parasites, and monogenetic flukes are entoparasites, and digenetic flukes and cestodes are endoparasites. As an external group in the analysis, we used mollusk sequences from the genomes of the Pacific oyster (Crassostrea gigas), the sea saucer (Lottia gigantea) and the Philippine mussel (Modiolus philippinarum), since it is known that mollusks are a sister group to flatworms (Bernhard et al., 2015; Laumer et al., 2015). The amino acid sequences of mollusks were taken from the MolluskDB 2.0 database (Caurcel et al., 2021). The genome identifiers of flatworms and mollusks, species names of organisms and their types, and lifestyle are presented in Supplementary Material 4.

**Statistical processing of the results.** To assess the presence of phospholipases of various orthologous groups in flatworms, for large taxa (Digenea, Cestoda, Monogenea and Turbellaria), we estimated the average number of phospholipase sequences for the orthogroup in the genome (n) and the standard deviation (σ). The average number n of sequences in each orthogroup by taxa shows how common phospholipase sequences are in the studied organisms. The standard deviations σ show a variation in the values of the number of sequences around the average. The greater the standard deviation, the greater the diversity in the number of sequences across taxa. Additionally, we evaluated the parameter f (representation, %), the fraction of organisms in a large taxon that contain at least one of the orthogroup sequences. If it is equal to 100 %, then all organisms of the taxon contain at least one sequence from the orthogroup. If some organisms do not contain any sequence from the phospholipase orthogroup, then the f value is less than 100 %.

## Results

As a result of the analysis carried out using the OrthoDom pipeline, 11 orthogroups were identified in flatworms, which contain reference sequences of phospholipases A2. Note that of all the groups of phospholipases A2, the sequences of which were used as a reference, only the sequences of group 9 did not show homology in the proteomes of mollusks and flatworms (they were not included in any of the orthogroups defined for these organisms). Thus, according to the classification of M. Murakami et al. (2020), 12 out of the 13 known groups of animal phospholipases A2 fell into the PLA2 orthogroups of mollusks and flatworms.

The Table shows the distribution of the identified orthogroups containing phospholipase sequences of flatworms and mollusks, and a number of statistical characteristics for them in terms of representation in the five main taxa. It can be seen that the correspondence between orthogroups and known types of phospholipases is non-exclusive. The Table shows that some orthogroups include several types of phospholipases. For example, the OG0003047 orthogroup contains sequences of phospholipase groups 1, 2, 5, 10. On the other hand, some types of phospholipases were represented by several orthogroups. For example, the sequences of phospholipase A2 group 6 split into orthogroups OG0000019, OG0000217, OG0000961. In other cases, each orthogroup corresponded to one type and group of PLA2.

**Table 1.**
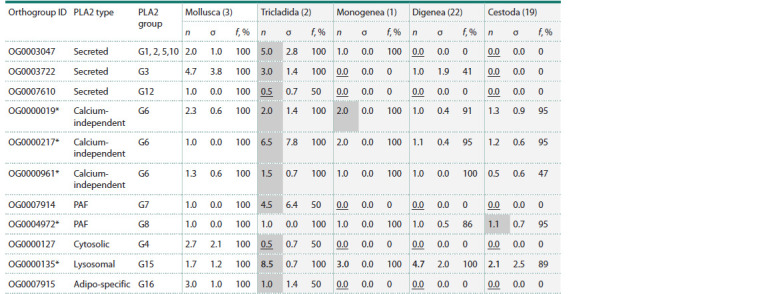
Characteristics of the occurrence of phospholipase A2 orthogroup genes in mollusks and large flatworm taxa Note. The rows correspond to different orthogroups of phospholipases. The columns include: the orthogroup ID, the type and groups of phospholipases represented in it; statistics for the studied large taxa (average values (n), standard deviations (σ) of the number of sequences in orthogroups by taxa, the representation (f) of sequences in different species). The number of species is given in parentheses next to the name of the taxon. The maximum average values of the n number of sequences in orthogroups by taxa are shown in bold, the minimum values are underlined. The largest n values for ortogroups are highlighted by gray background. The complete table is presented in Supplementary Material 5. *Orthogroups, the sequences of which are represented in all large taxa of flatworms.

First, it should be noted that orthogroups differ in the number of sequences they are represented by. Thus, in the orthogroup OG0000135, which represents the only group of lysosomal phospholipases group G15, the average number of orthologs per proteome in each taxon of flatworms is the largest, compared with other orthogroups (from 2.1 in cestodes to 8.5 in triclads). Note that in mollusks, this group of phospholipases is not the largest one: the average number of sequences per proteome is 1.7. This taxon has the most numerous OG00003722 group: the average number of sequences is 4.7 (secreted PLA2 G3).

The Table also shows that a high average number of proteome sequences assigned to different phospholipases is characteristic of Tricladida, which are free-living, in contrast to the other taxa, which are parasitic. Only in the case of orthogroup OG00004972 (type PAF, group G8), the average number of sequences per proteome in free-living worms (1) is less than in cestodes (1.11), but this number is not less than in the other taxa.

The Table also demonstrates that the orthogroups we have identified are unevenly represented in various taxa. First, the PLA2 groups, which are found in all large taxa of flatworms. These are calcium-independent type PLA2, namely orthogroups OG0000019, OG0000217, OG0000961 (the sixth group of PLA2). At the same time, proteins of the first two orthogroups are represented by the vast majority of species from large taxa (more than 90 %). Orthogroup OG0000961 is characterized by the absence of orthologs for half of the cestode species. For one of these groups (OG0000217), the average number of proteins in free-living worms (6.5) is several times higher than that in parasitic worms (1.1–2). Proteins of this group in cestodes are represented in only half of the studied species (the average number of PLA2 per proteome is 0.5, the standard deviation is 0.6).

Another orthogroup, the representatives of which are found in all taxa of flatworms, is OG0004972 (the eighth group of platelet-activating type PLA2). In all major taxa, these proteins are present in more than 95 % of species, except for the digenetic flukes, in which this proportion is 87 %. These genes have 1–2 copies per proteome.

Another orthogroup represented in all major taxa is OG0000135, which includes lysosomal PLA2 of group G15. The sequences of this group are represented by more than one copy per proteome, and are characterized by the largest number of copies compared to others (see above).

Secondly, in the Table, orthogroups specific to free-living worms can be distinguished, the genes of which are completely absent in parasitic worms. These orthogroups were divided into four types: secreted, PAF, cytosolic and adipo-specific (OG0007610, OG0007914, OG0000127, OG0007915, respectively). Proteins in all these orthogroups are present in at least one of the two free-living species studied by us.

Thirdly, the Table demonstrates the presence of orthogroups specific to individual parasitic taxa. For example, orthogroup OG0003047 (phospholipases of groups G1, G2, G5, G10) is found only in Monogenea (in all species). Orthogroup OG0003722 is found only in Digenea (about half of the species). At the same time, cestodes have the smallest number of phospholipase orthogroups, in particular, all secreted phospholipases are missing.

Thus, the results allow us to conclude that most of the animal PLA2 groups (12 out of 13) are found in free-living worms, and most of them have a large number of copies. The number of genes in orthogroups and the number of orthogroups in parasitic worms is reduced in comparison with the free-living ones. Monogenea have one orthogroup including secreted proteins, all calcium-independent, one orthogroup including PAF, and one including lysosomal phospholipases A2. In Digenea, proteins from an orthogroup other than Monogenea and orthogroups including PAF and lysosomal phospholipases are present. All calcium-independent PLA2, PAF, and lysosomal phospholipases A2 are present in cestodes, but the secreted ones are completely absent. Various taxa of parasitic worms have phospholipases common to all of them, as well as specific ones.

The structural diversity of phospholipases

**Fig. 2.**
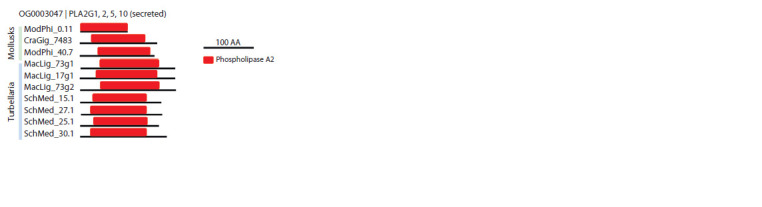
Domain structure of sequences of orthogroup OG0003047, phospholipase A2 of the secreted type. The scale corresponding to 100 amino acids is shown on the right, the phospholipase domain is marked in red. The figure shows 10 sequences randomly selected among all the sequences of the OG0003047 orthogroup.

**Fig. 3.**
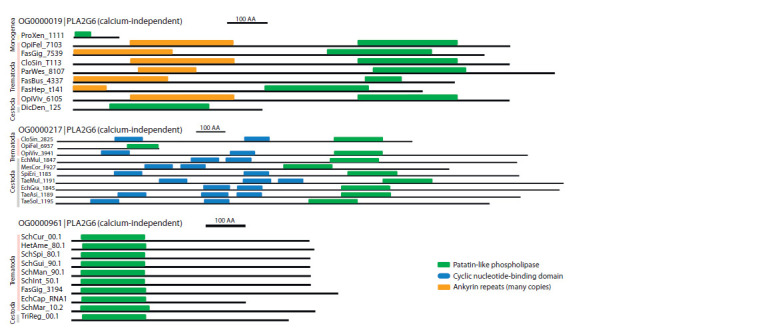
Domain structure of sequences of orthogroups OG0000019, OG0000217, OG0000961, calcium-independent phospholipase A2. The scale corresponding to 100 amino acids is shown on the right, the patatin-like phospholipase domain is marked in green, the cNMP domain is blue, and ankyrin repeats are orange. The figure shows 10 sequences (from 30 in total) randomly selected among all the sequences of orthogroups OG0000019, OG0000217, OG0000961.

The domain organization for a number of phospholipases is shown in Figures 2 and 3. Figure 2 shows the domain structure of phospholipases from the OG0003047 orthogroup, which includes the reference proteins of the PLA2 groups G1, G2, G5 and G10.

Figure 2 shows that the sequences of secreted phospholipase A2 orthogroup OG0003047 have a length of approximately 200–250 amino acids. The phospholipase domain occupies more than 80 % of the total protein. Thus, the primary structure of secreted PLA2 in flatworms shows high similarity with human PLA2 structures of the corresponding types (Turnaev et al., 2022). Note that this orthogroup is represented only in free-living organisms.

The domain organization of the sequences of orthogroups OG0000019, OG0000217 and OG0000961 is shown in Figure 3. These are enzymes that belong to group 6. Despite the fact that group 6 PLA2 has been divided into three specified orthogroups, all of them contain a patatin domain key to this group (Fig. 3). The domain structure of the sequences of the OG0000019 orthogroup corresponds to the subgroup A typical for group 6 PLA2, which is characterized by a patatin domain and seven ankyrin domains. The composition of the domains of the OG0000217 orthogroup proteins corresponds to the typical group 6 subgroup C PLA2, which in addition to the patatin domain has three cNMP domains. The composition of the sequence domains of the OG0000961 orthogroup is similar to subgroups D and E typical for group 6 PLA2, which are characterized only by a patatin-like phospholipase domain located at the N-end of the sequence (Turnaev et al., 2022).

Thus, the analysis of the functional domains of phospholipases shows that proteins belonging to phospholipases of different types, but having a similar domain composition, form a common orthogroup, and sequences with different domain compositions of phospholipases of even the same type break down into different orthogroups.

Phylogenetic analysis of flatworm phospholipases

For orthogroups, the domain structure of which is presented in Figures 2 and 3, we reconstructed phylogenetic trees.

**Fig. 4.**
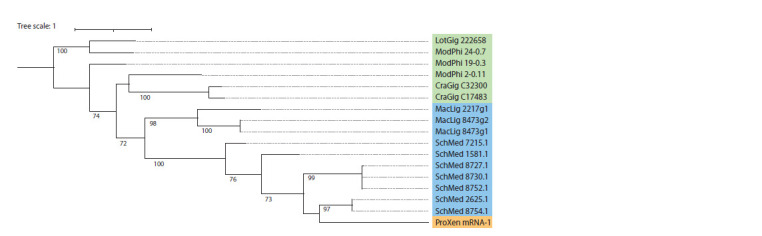
Phylogenetic tree of phospholipase A2 orthogroup OG0003047 (PLA2G1, 2, 5, 10, secreted). In the figure, the sequences of mollusks (Mollusca) are highlighted in green, free-living worms (Turbellaria), in blue, and monogenea (Monogenea), in orange.

Sequences of the OG0003047 orthogroup were found in free-living flatworms and one representative of Monoge- nea (Fig. 4). In species of free-living flatworms, the number of sequences belonging to this orthogroup is high (see the Table), in a representative of Monogenea species, Protopolystoma xenopodis (short designation ProXen), only one gene encoding a phospholipase of this type is observed.

Phylogenetic trees of orthogroups containing PLA2 of group 6 are presented in Supplementary Material 6. In the figures of calcium-independent PLA2 of group 6 (Supplementary Material 6, Fig. 1–3), similar patterns can be seen. It is worth noting that protein sequences of parasitic flatworms are highly conservative. In Figure 3, it can be seen that the domain structure of the sequences is similar among representatives of different parasitic taxa. This allows us to conclude that group 6 PLA2 is a conservative protein that plays a key role in the basic processes of life of parasitic flatworms.

**Fig. 5.**
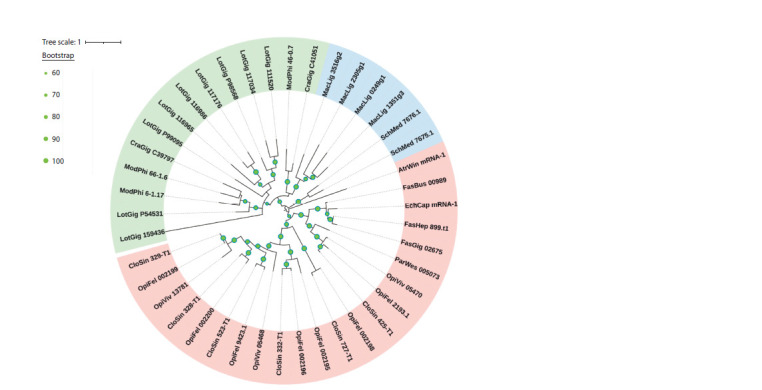
Phylogenetic tree of sequences of phospholipases A2 of orthogroup OG0003722 (PLA2 G3, secreted). In the Figure, mollusks (Mollusca) are highlighted in green, free-living flatworms (Turbellaria), in blue, and digenetic flukes (Digenea), in red.

Secreted phospholipases A2, which belong to orthogroup OG0003722, are worth noting. This orthogroup is characterized by the fact that in parasitic worms only the Digenea contains sequences of this orthogroup. The phylogenetic tree of sequences belonging to this orthogroup is shown in Figure 5.

Figure 5 shows that several copies of the PLA2 gene of this orthogroup are found in free-living worms. Digenetic flukes also have several copies of this gene, which are distributed in different clades. This suggests that duplications of the PLA2 gene of the G3 group are characteristic of these organisms. As a rule, the molecular evolution of parasites proceeds much faster compared to representatives of free-living organisms (Trouvé et al., 1998). The analysis of phylogenetic trees confirms this statement for phospholipase A2, where longer branches are observed in parasites, which indicates a high rate of evolution of these molecules

## Discussion

Despite the fact that phospholipases of various types, PLA2 among them, are components of ESP of parasitic flatworms (Wang et al., 2014) and that an association with carcinogenesis in the host has been demonstrated for a number of them (Hu et al., 2009; Shang et al., 2017), they are still insufficiently studied for the Platyhelminthes taxon (Dennis et al., 2011). Here, almost all known groups of phospholipases in flatworms were identified. The OrthoDom pipeline allowed to split them into orthogroups, taking into account the domain structure. These results are consistent with the classification of phospholipases A2 and their domain organization, presented in the works of E.A. Dennis et al. (2011). The method of identifying orthologs based on the domain structure has demonstrated its effectiveness in isolating orthogroups of proteins, taking into account the differences in the composition of their domains

Our analysis made it possible to identify them and showed that in the evolution of A2 phospholipases in flatworms, peculiarities can be identified that are characteristic of the evolution of parasite genomes, for example, gene loss due to a parasitic lifestyle (Langleib et al., 2024). Indeed, our study demonstrated that some PLA2 groups are reduced in parasitic flatworms, and most genes are represented by a single copy. There are groups of phospholipases lost in some large taxa.

Comparative analysis of orthogroups of PLA2 genes shows that a relatively high degree of duplication is observed among PLA2 in free-living worms, with an average number of paralogs per species reaching five. This phenomenon implies the presence of significant adaptive capabilities, which may be due to a variety of environmental factors. Free-living organisms exposed to higher levels of environmental competition can use this diversity of PLA2 genes to increase viability and resistance to environmental changes. However, in orthogroup OG0000135 containing lysosomal type PLA2, genes are duplicated even in parasitic flatworms. What caused this anomaly remains to be seen

A number of experimental studies have shown that some phospholipases A2 can participate in carcinogenesis, contributing to the activation of a number of cellular signaling pathways and interaction with the host body’s immune system. For example, chronic infection caused by C. sinensis leads to liver fibrosis and cholangiocarcinoma (Shang et al., 2016). Moreover, C. sinensis uses group 3 phospholipases A2 as an ESP, which plays an important role in host kidney pathogenesis (Wu et al., 2021). As a result of the study, it was found that among the secreted phospholipases of digenetic flukes, only phospholipase A2 of group 3 is present, whereas in cestodes there are no secreted phospholipases A2. Given that parasitic flatworms are able to manipulate the metabolism of their hosts by using phospholipases to extract the necessary resources, it can be assumed that similar mechanisms may work in cancer cells.

## Conclusion

Phospholipases A2 are a family of hydrolases that catalyze the hydrolysis of phospholipids, playing a key role in many molecular processes in the functioning of cells and the body as a whole. Their diversity in flatworms has been poorly studied, and in our work, we conducted such an analysis for the first time. We found that 12 out of the 13 known types of phospholipases A2 are present in free-living worms. These organisms have an increased number of gene copies compared to parasitic worms. Unique features of some orthogroups have been identified, which may probably be associated with carcinogenesis in the host caused by a parasitic infection

## Conflict of interest

The authors declare no conflict of interest.
